# Detecting Subtle Shifts in Ecosystem Functioning in a Dynamic Estuarine Environment

**DOI:** 10.1371/journal.pone.0133914

**Published:** 2015-07-27

**Authors:** Daniel R. Pratt, Andrew M. Lohrer, Simon F. Thrush, Judi E. Hewitt, Michael Townsend, Katie Cartner, Conrad A. Pilditch, Rachel J. Harris, Carl van Colen, Iván F. Rodil

**Affiliations:** 1 National Institute of Water and Atmospheric Research, P.O. Box 11–115, Hillcrest, Hamilton, New Zealand; 2 Institute of Marine Science, University of Auckland, Private Bag 92019, Auckland, New Zealand; 3 Department of Biological Sciences, University of Waikato, Hamilton, New Zealand; 4 Marine Biology Group, Biology Department, Ghent University, Krijgslaan 281-S8, Ghent, Belgium; 5 CIIMAR, Interdisciplinary Centre of Marine and Environmental Research, University of Porto, Rua dos Bragas 289, Porto, Portugal; CSIR- National institute of oceanography, INDIA

## Abstract

Identifying the effects of stressors before they impact ecosystem functioning can be challenging in dynamic, heterogeneous ‘real-world’ ecosystems. In aquatic systems, for example, reductions in water clarity can limit the light available for photosynthesis, with knock-on consequences for secondary consumers, though in naturally turbid wave-swept estuaries, detecting the effects of elevated turbidity can be difficult. The objective of this study was to investigate the effects of shading on ecosystem functions mediated by sandflat primary producers (microphytobenthos) and deep-dwelling surface-feeding macrofauna (*Macomona liliana*; Bivalvia, Veneroida, Tellinidae). Shade cloths (which reduced incident light intensity by ~80%) were deployed on an exposed, intertidal sandflat to experimentally stress the microphytobenthic community associated with the sediment surface. After 13 weeks, sediment properties, macrofauna and fluxes of oxygen and inorganic nutrients across the sediment-water interface were measured. A multivariate metric of ecosystem function (MF) was generated by combining flux-based response variables, and distance-based linear models were used to determine shifts in the drivers of ecosystem function between non-shaded and shaded plots. No significant differences in MF or in the constituent ecosystem function variables were detected between the shaded and non-shaded plots. However, shading reduced the total explained variation in MF (from 64% in non-shaded plots to 15% in shaded plots) and affected the relative influence of *M*. *liliana* and other explanatory variables on MF. This suggests that although shade stress may shift the drivers of ecosystem functioning (consistent with earlier investigations of shading effects on sandflat interaction networks), ecosystem functions appear to have a degree of resilience to those changes.

## Introduction

Many human activities result in stress and disturbance to natural ecosystems. Sometimes the effects of stress and disturbance are distinct and readily observable; at other times, the effects are more subtle [1–4]. Detecting the effects of stressors before they have caused widespread ecological community change and loss of ecosystem services is a priority for resource managers, as remedial actions are more likely to be effective at this stage. However, identifying the effects of stressors before major changes have occurred can be challenging in dynamic, heterogeneous ‘real-world’ ecosystems, particularly for response variables that are difficult to measure precisely [5–7].

Changeable environmental conditions (e.g. light, temperature, wind and current patterns) may conceal ecological patterns and hinder our ability to track responses to low levels of stress using conventional measures of ecosystem functioning [8], [9]. Furthermore, the effects of stressors can occur via indirect mechanisms [10], for example, by disrupting positive feedbacks between organisms and local habitat characteristics. Thus, analysing relationships between drivers (e.g. the types, densities and diversity of species) and processes (e.g. primary production, nutrient regeneration) can be effective for tracking the effects of subtle stressors, as opposed to testing for differences in the magnitudes of the processes per se [11]. This approach not only recognises that ecosystem functions are underpinned by interaction networks connecting organisms and their physical environments in complex ways [12–14] but may provide a more sensitive way to identify threats posed by indirect effects in highly variable, real world systems.

The goal of this study was to investigate the potential for low levels of stress to influence ecosystem functions on a dynamic intertidal sandflat in New Zealand. It is relatively well established that the influence of species on ecosystem functions is determined by their densities, sizes and functional roles [14–19]. Variables used to describe ecosystem functioning on sandflats (most notably, fluxes of oxygen and nutrients across the sediment-water interface that provide information on community-integrated rates of primary productivity, oxygen consumption and nutrient release) often involve multiple species and many interrelated processes. For example, in sedimentary systems, organic matter is remineralised by aerobic and anaerobic bacteria into inorganic nutrients, which are taken up and used by oxygen-producing photosynthesisers to generate more organic matter [20]. Simultaneously, foraging animals bioturbate sediments, produce organic rich biodeposits and faeces, and excrete inorganic nutrients (e.g. [16], [21–23]). Thus, analysing ecosystem responses with consideration to the many complicated sets of interacting processes would be an advance.

Here, we investigated a system of sandflat interactions that centred on an infaunal deposit-feeding bivalve, *Macomona liliana*, and populations of microalgae that live in surface sediments, microphytobenthos (MPB). MPB are the principal primary producers on dynamic, unvegetated coastal sandflats [24] and play a major role in fuelling benthic food webs [20], [25]. Adult *M*. *liliana* live at a depth of 2–10 cm in mid-shore sandy sediments and feed on surface MPB using an inhalant siphon [26]. During feeding, this species creates pore-water pressure gradients that oxygenate deeper sediments and force nutrient rich pore-water upwards towards the sediment surface, thereby potentially facilitating MPB production [27–29]. *M*. *liliana* form dense beds over large areas and are common to intertidal sandflats in New Zealand’s North Island. Furthermore, tellinid bivalve species are common around the world and many exhibit similar behaviours [28]. Generally, interactions between bioturbating macrofauna and MPB are well-studied because they have a profound effect on the structure of sediments and macrofaunal communities [29], [30] and on primary production, nutrient cycling [22], [31], and sediment transport processes [32].

Considering the importance of MPB for benthic functioning and food webs, stressors that limit MPB may have widespread and cascading consequences for community processes and macrofaunal populations. Nevertheless, field studies that focus on indirect stressor effects on ecosystem processes are rare. In this study, shade cloths were deployed in the field to experimentally reduce sunlight availability at the sediment surface to stress the MPB community; decreased light levels are an important consequence of elevated water turbidity [33]. This work was nested within a larger experiment that tested the effects of multiple stressors on the interaction strengths between large bioturbating macrofauna, nutrient stocks and MPB biomass [34]. The focus here was to determine how shade-stress may indirectly alter rates of ecosystem functions and the importance of large *M*. *liliana* individuals as drivers of ecosystem functioning. Therefore, we assessed the contribution of *M*. *liliana* densities and sizes in conjunction with a range of physical (sediment properties, nutrient stocks) and biological (species abundances, diversity) co-variables. We analysed a multivariate metric of ecosystem function (hereafter MF) because multivariate data has been shown to be highly sensitive to changes in stressors relative to individual univariate measures of ecosystem function [35]. Moreover, the integrity of ecosystems and their ability to deliver goods and services that benefit society [36] is underpinned by multiple interrelated functions that may respond to stress in different ways [35], thus MF provides a more integrated indicator of shifts in functioning that relate to service delivery.

## Materials and Methods

### Ethics statement

This study complied with all existing legislation governing animal welfare and field-based experiments. Animal ethics approval/permits were not sought as benthic invertebrate fauna manipulated/sampled in this study are exempt from the Animal Welfare Act 1999. Permission to conduct the field experiment was obtained from Auckland Regional Council (CN37056:FN12876).

### Study area

Manukau Harbour is a tidally dominated (mean tidal range = 2.8 m) system connected to the Tasman Sea on the west coast of the North Island of New Zealand. The experiment was conducted within a ~800 x ~350 m area of intertidal sandflat at Wairoa Island (37° 01.3'S; 174° 49.2'E). Sediment properties at the study site (median grain size = 142–210 μm, <2% mud particles <63 μm in size) are typical of New Zealand intertidal sandflats influenced by wind-waves and moderate-to-strong tidal currents. The macrofaunal community at the study site is typically dominated both numerically and in terms of biomass by bivalves, in particular *M*. *liliana* and to a lesser extent (in order of relative abundance), *Soletellina siliquens*, *Paphies australis* and *Austrovenus stutchburyi*. The other dominant macrofaunal group at the site was polychaetes, including *Aonides trifida*, *Scolelepis sp*., *Orbinia papilosa* and *Nicon aestuariensis*. There were relatively few crustaceans at the site, with only *Paracalliope novizealandiae* (an amphipod species) found at a density of >1 individual per 10 cm diameter sediment core.

### Experimental design

This study was based on a subset of treatments that were part of a larger experiment aimed at determining how loadings of nutrients and sediments from the catchment affect the ecological properties and functioning of sandflats in the receiving environment [34]. In the Thrush et al. (2014) [34] study, 3 levels of cover (shaded, bare sediment and “non-shaded” procedural controls) were used along with 3 *M*. *liliana* density treatments, 3 nutrient addition treatments and one ambient sediment treatment (a total of 196 plots spread across 7 experimental blocks). Here, however, the focus was more detailed and process-based, and entailed measurements that were more time- and gear-intensive (see benthic chamber incubation section below). We prioritised our effort on *M*. *liliana* density treatments in 6 blocks in the shaded and non-shaded procedural control plots only (36 of the 196 total plots).

Shade treatments were established using black shade cloths that were designed to reduce incident light levels. The shade cloths were suspended 15–20 cm above the experimental plots by attaching them to 4 m^2^ steel grids (mesh size = 150 × 150 mm) that were staked into the sediment at each corner. The purpose of shading was to reduce light levels in a system that already experiences moderate to high levels of turbidity; shade treatments were not expected to eliminate photosynthesis, but rather to produce changes in MPB and their associated ecosystem functions by temporarily elevating shade-stress. Non-shaded procedural controls (hereafter “non-shaded”) contained the steel grids without the shade cloths. Differences in seabed light intensity and temperature between shaded and non-shaded plots were quantified by deploying HOBO loggers and Thermochron i-buttons. To manipulate the densities of *M*. *liliana*, plots (1 m^2^) positioned in the centre of the larger 4 m^2^ treatment areas were excavated to 18 cm depth and sediments were sieved on 10 mm mesh to remove shell hash and large macrofauna. Subsequently, the sieved sediments were returned to the excavated plots and three specific densities of large *M*. *liliana* (0, 50 and 200 individuals >20 mm shell length per m^2^) were replanted back into the plots, evenly spread within the central 1 m^2^ area (background density in the study site ranged between ~80–144 individuals >20 mm shell length per m^2^). We anticipated that mortality and movement of individuals across plot boundaries would lead to changes in *M*. *liliana* densities over time, with the treatments ultimately producing a gradient in *M*. *liliana* density rather than fixed density categories. At the end of the experiment, *M*. *liliana* densities ranged from ~16 to 252 individuals per m^2^; there was no evidence (e.g. presence of empty shells) from field or laboratory (core analysis) observations to suggest the occurrence of notable mortality in replanted *M*. *liliana*. The experiment was established on 25–29 October 2011. Responses were measured (solute fluxes in benthic incubation chambers, see next section) approximately 13 weeks after treatment establishment on 25–26 January 2012. Shade cloths were removed from the plots prior to the assessment of treatment effects, as the aim was to capture the legacy effects of shading on key system responses (such as benthic primary productivity rates) and not the immediate effects of light reduction. The sampling of the experimental blocks was divided across two consecutive days, with blocks 2, 3 and 5 sampled on January 25^th^, and blocks 1, 4 and 6 sampled on January 26^th^. Fluxes were measured during mid-day high tide periods (11:00–15:00) under sunny, calm conditions. Mean sunlight intensity during incubations was 21,082 and 19,843 lux on days 1 and 2, respectively, and ambient seawater temperature was 22.9 and 23.5°C.

### Benthic chamber incubations

Ecosystem functioning was evaluated based on fluxes of dissolved oxygen and nutrients (ammonium nitrogen, NH_4_^+^; nitrate + nitrite nitrogen, NO_X_; dissolved reactive phosphorus, DRP) across the sediment-water interface. Fluxes of oxygen and nutrients are indicative of ecological processes critical to system functioning such as photosynthetic oxygen production, remineralisation of organic matter, and inorganic nutrient uptake by algae and microbes. Sunlit and darkened chambers (0.85 l volume of seawater enclosed over a 0.016 m^2^ area of sediment; [37]), were deployed to quantify solute fluxes when photosynthesis was possible, and not possible, respectively.

On the incoming tide, when the plots were covered by c. 30 cm of water, chambers were rinsed with ambient seawater and carefully placed on the seabed with no air bubbles trapped inside. Sampling tubes were flushed with 150 ml of seawater before time = 0 samples (60 ml) were collected. After a high tide incubation period of c. 4 hr, final samples (60 ml) were collected from all chambers. Water column effects on chamber solute fluxes were accounted for by incubating seawater *in-situ* in paired light and dark bottles (n = 3) for the duration of the chamber incubation. Ambient bottom water samples (from outside the chambers) were also collected in conjunction with the initial and final samples. The exact times of chamber deployment and chamber water sampling were recorded in all cases. HOBO data loggers (n = 10 per day) were deployed 10 cm above the seabed in conjunction with chamber incubations to quantify variability in ambient water temperature and sunlight intensity (lux) that can influence sediment oxygen and nutrient exchange by altering the rates of biological and physico-chemical processes.

After collection, dissolved O_2_ concentrations in each water sample were measured as soon as possible using a calibrated optical dissolved oxygen probe. Water samples were then filtered through a 0.8 μm pore size glass fibre filter into sterile containers and stored on ice in the dark for transport to the laboratory, where they were frozen until analysis. Dissolved inorganic nutrient analysis was performed using standard methods for seawater on an Astoria-Pacific 300 series segmented flow auto-analyser (detection limit of 1 μg l^−1^ for N and P).

### Sampling of sandflat habitat characteristics

Sediments were sampled to determine macrofaunal abundances and sediment properties within the inner 1 m^2^ of each plot. The sediment properties measured included pore water nutrient concentrations, sediment grain size distribution, organic matter content, and algal pigment concentrations (chlorophyll-*a*, phaeophytin). The sampling methods for macrofauna and sediment properties are standard and were described in detail by Thrush et al. (2014) [34]. Additional information on meiofauna [38] and sediment erodibility [39] is also available from experimental plots at this site, though it is not discussed here.

Briefly, pore water nutrient samples from the upper 5 cm of the sediment column were collected from each plot in a reservoir with a water permeable (but not sediment permeable) membrane. The pore water was then filtered, stored and analysed for dissolved inorganic nutrients using the same methods as for the chamber incubated samples described above. Three cores of sediment (2.3 cm diameter, 2 cm deep) were collected from each plot and amalgamated for analyses of grain size, organic matter content (OC) and algal pigments. Macrofaunal data (total abundance, taxonomic richness, evenness, diversity, and individual species abundances) were determined by averaging three larger cores of sediment (10 cm diameter, 10 cm deep) collected from each plot and sieved across a 0.5 mm mesh screen. Specimens were preserved in 70% isopropyl alcohol solution with rose bengal, and identified to the lowest possible taxonomic level. The remaining sediment within the central 0.25 m^2^ was excavated to a depth of about 15 cm and sieved on a coarser 10 mm mesh screen to quantify densities of large bivalve individuals (*M*. *liliana* and *A*. *stutchburyi*).

### Data analysis

Fluxes measured in the incubation chambers were used to evaluate key processes indicating ecosystem functions: sediment oxygen consumption, nutrient regeneration and uptake, and primary production (Table 1). Sediment oxygen consumption and gross nutrient efflux were determined in the dark (in the absence of oxygen production and nutrient uptake by photosynthesising MPB; [40]). Photosynthetic uptake of nutrients was calculated from the difference between nutrient fluxes in darkened and sunlit chambers. Gross primary production was determined from the rate of oxygen production in sunlit chambers minus oxygen consumption in darkened chambers. Rates of gross primary production per unit of chlorophyll-*a* were calculated as a measure of the sediment’s photosynthetic efficiency. Rather than analysing these responses individually, all of the ecosystem function variables listed in Table 1 were combined in a resemblance matrix generated from between-sample similarities (Euclidean distances) in PRIMER (v.6) to derive a multivariate metric of ecosystem function (MF) to use as a response variable in subsequent analyses. This follows the approach of Villnäs et al. (2013) [35]. All subsequent analyses were conducted in PERMANOVA add-on in PRIMER (v.6; [41]).

**Table 1:** Ecosystem functions assessed from solute flux data and later used to create a multivariate metric of ecosystem functioning (MF).

Ecosystem function		Abbrev.	How derived	Median flux (min–max)			
				Non-shaded		Shaded	
*Primary production*							
1)	Net Primary Production	NPP	Light O_2_ flux	702.7	(−241.1–2462.9)	1119.3	(−263.0–2687.3)
2)	Gross Primary Production	GPP	Light O_2_ flux–Dark O_2_ flux	2007.9	(169.4–4110.3)	1921.3	(−61.6–3891.3)
3)	Biomass normalised GPP	GPP_Chl-a_	(Light O_2_ flux–Dark O_2_ flux)/Chl-*a*	160.5	(14.2–308.2)	193.1	(−4.2–421.6)
*Community metabolism*							
4)	Sediment oxygen consumption	SOC	Dark O_2_ flux	1201.9	(246.5–2439.9)	1142.4	(231.1–2148.1)
*Nutrient regeneration and uptake*							
5)	Net nutrient efflux	Net NH_4_^+^	Light NH_4_^+^ flux	43.1	(−22.5–161.5)	35.4	(−9.5–156.9)
6)		Net NO_X_	Light NO_X_ flux	−9.9	(−15.5 to −4.4)	−9.5	(−47.6 to −4.9)
7)		Net DRP	Light DRP flux	1.8	(−28.3–20.3)	0.9	(−2.4–13.1)
8)	Gross nutrient efflux	Gross NH_4_^+^	Dark NH_4_^+^ flux	41.9	(−103.4–563.2)	55.9	(7.9–200.7)
9)		Gross NO_X_	Dark NO_X_ flux	−9.6	(−16.1 to −1.1)	−8.9	(−16.7 to −1.8)
10)		Gross DRP	Dark DRP flux	6.1	(−31.4–49.0)	4.7	(−0.9–26.8)
11)	Nutrient uptake	NH_4_^+^ up	Dark NH_4_^+^ flux − Light NH_4_^+^ flux	13.2	(−132.8–401.6)	27.8	(−67.7–81.9)
12)		NO_X_ up	Dark NO_X_ flux − Light NO_X_ flux	2.0	(−5.3–6.8)	0.1	(−7.3–41.5)
13)		DRP up	Dark DRP flux − Light DRP flux	4.4	(−31.4–40.9)	3.4	(−7.3–21.8)

Ecosystem function response variables are expressed in units of μmol m^−2^ h^−1^

As a first step in the analysis, one-way PERMANOVA tests were performed to determine differences in sediment properties (e.g. mud content, pore water nutrient concentration), MF and constituent ecosystem function measures between non-shaded and shaded plots. These were followed by factorial PERMANOVA models that tested for effects of shading and large *M*. *liliana* density treatments (fixed factors) in combination with sampling block (random factor). ANOSIM was used to assess variation in species abundances between non-shaded and shaded plots.

However, the primary goal of the analysis was to compare the drivers of variation in MF in shaded and non-shaded plots; this was accomplished by dividing the full dataset and analysing the shaded and non-shaded data separately. Preliminary checks of the data suggested that there were no systematic differences in sediment properties between shaded and non-shaded treatments (also see supplementary material for Thrush et al. 2014, http://dx.doi.org/10.1890/13-1879.1). Distance-based linear models (DistLM) were then developed to identify predictor variables that best describe patterns in MF in the non-shaded and shaded treatments. All predictor variables were standardised to range between 0 and 1 prior to analysis. Initially, significant predictors of MF were identified when fitted individually in marginal tests using shade and non-shade data. Then, the best set of explanatory variables for MF were determined using a backwards elimination procedure, which starts with a large suite of explanatory variables and proceeds to remove individual variables sequentially (sequential tests). Decisions on whether to retain or remove variables at each step were based on the corrected Akaike’s Information Criterion (AIC_c_) scores, whereby retained variables were always significant at *p* < 0.15 and explained >10% of the total variance. AIC_c_ was the most suitable selection criteria given the high number of explanatory variables available, relative to the number of samples [41]. The relative proportion of variance explained by predictor variables in isolation, and when combined, was also calculated (‘variance partitioning’ following [42], [43]).

## Results

### Shading effects on benthic ecosystem properties

Mean daytime light intensity reaching the seabed in the 3 days prior to sampling was ~8 times higher in the non-shaded compared to shaded plots. However, the differences between these treatments were greater at low tide (non-shaded: 84,452 lux; shade: 9005 lux) relative to when the plots were submerged (non-shaded: 29,258 lux; shaded: 4373 lux) [39], reflecting the influence of water column depth and suspended sediments on light attenuation at high tide. Mean temperature during the same time frame (for high and low tide periods combined) was also slightly lower in shaded (21.9°C) compared with non-shaded plots (24.4°C).

**Fig 1:**
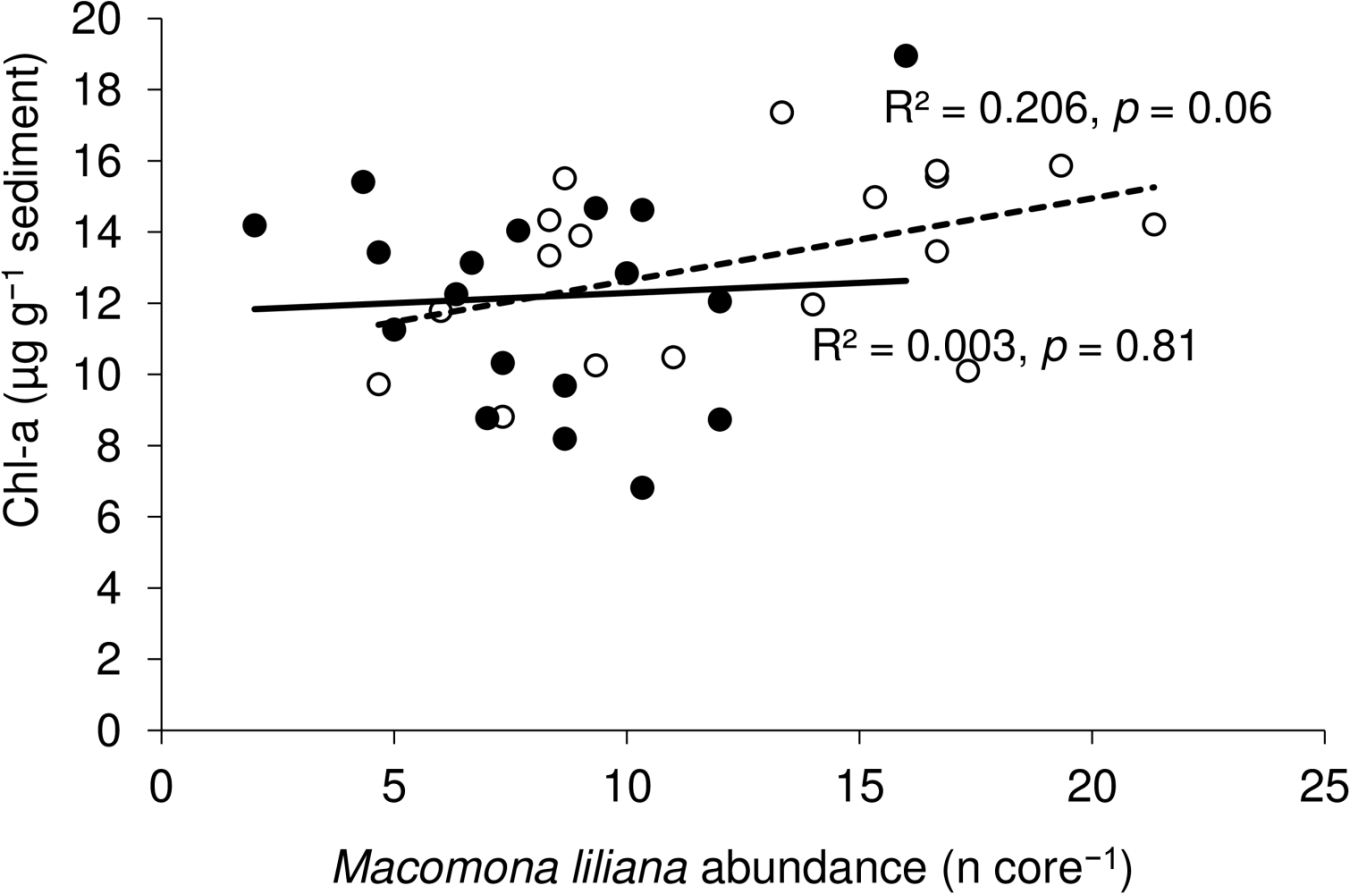
Relationships between M. liliana and MPB in non-shaded (open circles) and shaded (black circles) plots.

**Fig 2:**
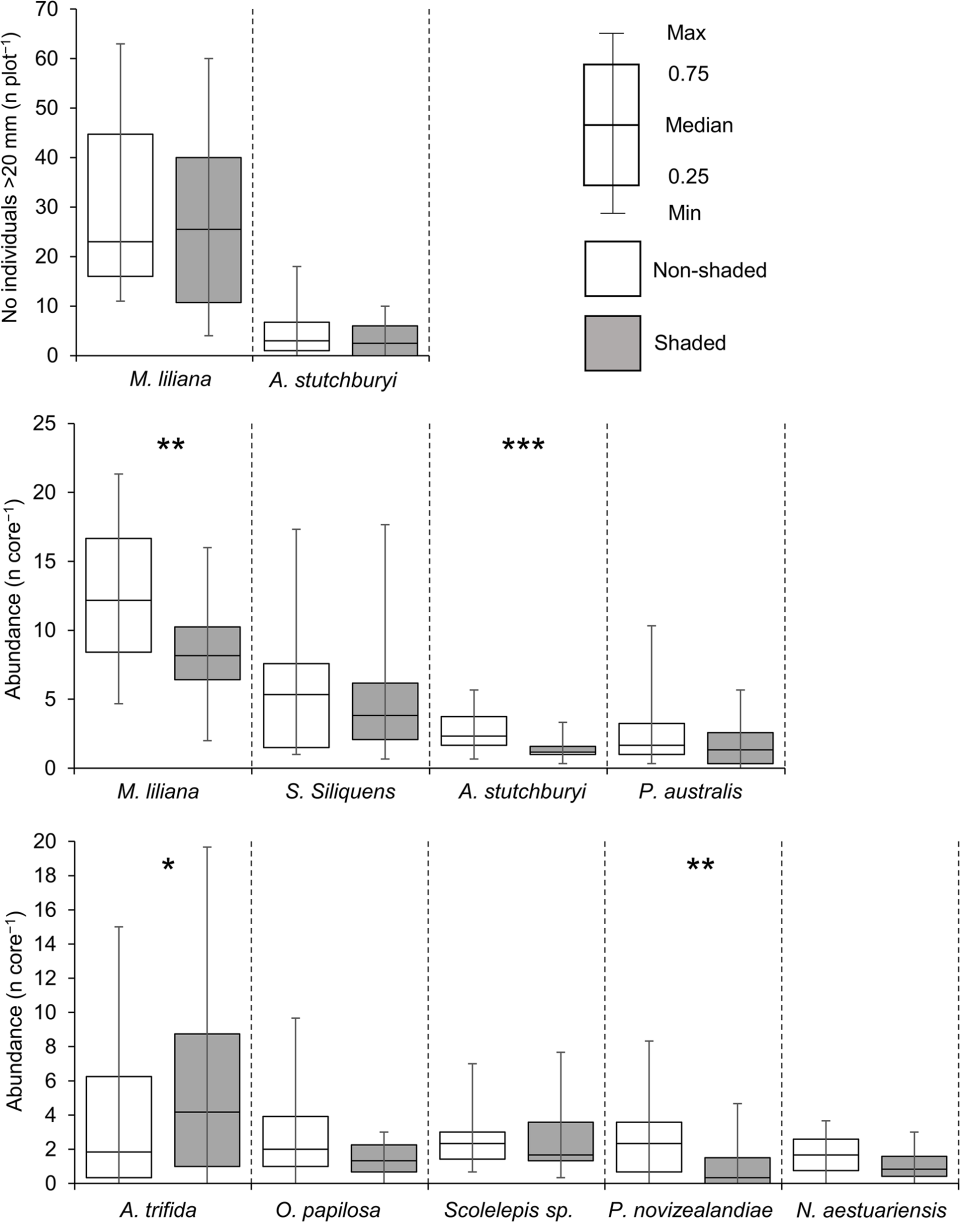
Changes in the abundances of large (> 20 mm) bivalves (middle panel), bivalves including all size classes measured in cores (middle panel) and other macrofauna (four polychaetes and one amphipod, bottom panel) between non-shaded (open) and shaded (grey bars) treatments. Asterisk denotes significance treatment effects (unpaired t-tests) at p <0.05 (*), <0.01 (**) and <0.001 (***).

**Fig 3:**
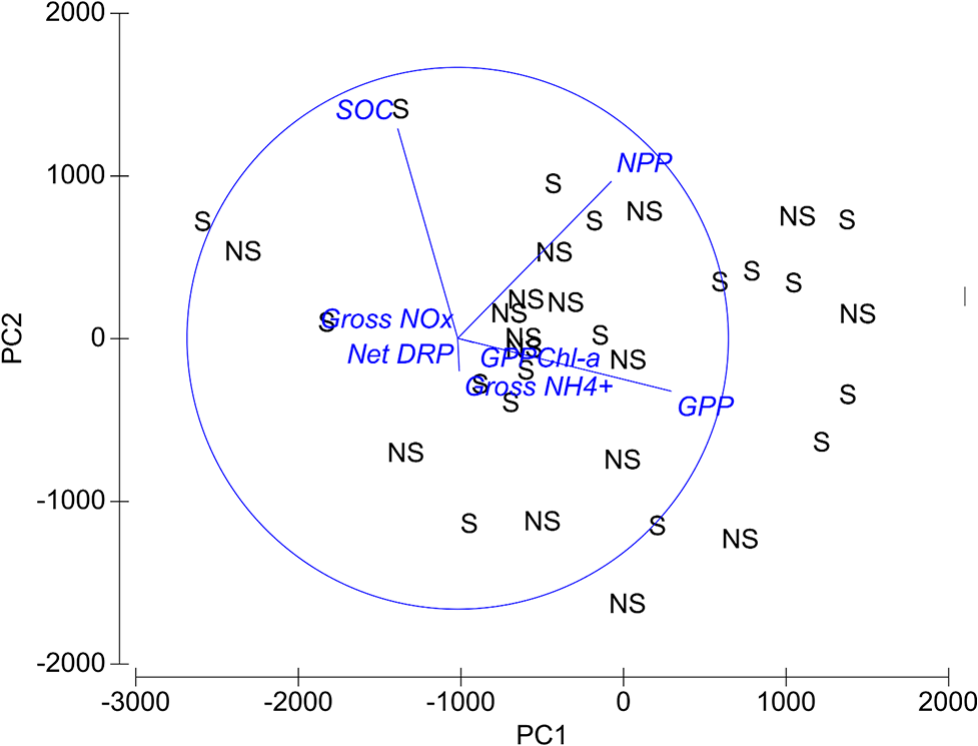
PCA ordination showing changes in macrofauna community composition between non-shaded and shaded treatments. All 51 species were included in analysis. Vector overlays show nine of the most influential species (At = *Aonides trifida*, As = *Austrovenus stutchburyi*, Ml = *Macomona liliana*, Na = *Nicon aestuariensis*, Op = *Orbinia papilosa*, Pa = *Paphies australis*, Pn = *Paracalliope novizealandiae*, Sc = *Scolelepis* sp., Ss = *Soletellina siliquens*, the rest omitted to improve figure clarity).

For most of the sediment properties (including pore water nutrients), no significant treatment (shade vs non-shade) effects were detected (Table 2). Chlorophyll-*a* displayed large variation (with a range of >8 μg g^-1^ sediment) in both shaded and non-shaded plots (Table 2) and no effect of treatment was detected (one-way PERMANOVA, *p* > 0.28). Relationships between *M*. *liliana* and chlorophyll-*a* have been demonstrated in previous studies [44], [45]; here, they were positively correlated in the non-shaded treatments, but no significant relationship was detected in the shaded treatments (Fig 1). Between treatment differences in densities of large (>20 mm), adult *M*. *liliana* measured at the plot scale (0.25 m^2^) were also not detected (shade vs non-shade, *p* = 0.38; Fig 2). However, *M*. *liliana* densities assessed from macrofaunal cores (which included adults as well as juveniles down to 0.5 mm shell width) declined significantly in the shaded plots (*p* < 0.01). The most notable effect of shading was a 22% decline in macrofaunal community abundance from ~48 to 35 individuals per 0.0079 m^2^ core (*p* < 0.01) and a shift in macrofaunal community composition (ANOSIM, *p* = 0.015; Fig 3). This was largely driven by reductions in the abundances of *M*. *liliana* juveniles and three other species (*A*. *stutchburyi*, *O*. *papillosa* and *P*. *novizealandiae*; Fig 2).

**Table 2:** Sediment properties for non-shaded and shaded treatment plots.

Variable	Units	Median (min—max)	
		Non-shaded	Shaded
MGS	μm	178 (142–208)	172 (147–210)
Mud	% (< 63 μm)	0.46 (0.05–1.61)	0.37 (0.02–1.18)
OC	%	1.00 (0.80–1.21)	0.80 (0.70–1.12)
Chl-*a*	μg g^−1^ sediment	13.7 (8.81–17.4)	12.6 (6.81–19.0)
Phaeo	μg g^−1^ sediment	3.59 (1.02–10.6)	5.70 (0.71–14.0)
pw-NH_4_^+^	μmol L^−1^	82.8 (41.7–127.8)	88.2 (43.2–178.5)
pw-NO_X_	μmol L^−1^	0.75 (0.50–2.00)	0.79 (0.64–2.86)
pw-DRP	μmol L^−1^	8.22 (2.42–12.8)	7.99 (3.55–17.4)

MGS = median grain size (μm), Mud = mud content (% [>63 μm]), OC = organic content (%), Chl-*a* = chlorophyll-*a* content (μg g^−1^ sediment), Phaeo = phaeophytin (μg g^−1^ sediment), pw-NH_4_^+^ = pore-water NO_4_^+^ concentration (μmols l^−1^), pw-NO_X_ = pore-water NO_X_ concentration (μmols l^−1^), pw-DRP = pore-water DRP concentration (μmols l^−1^)

### Shading effects on multivariate ecosystem function

**Fig 4:**
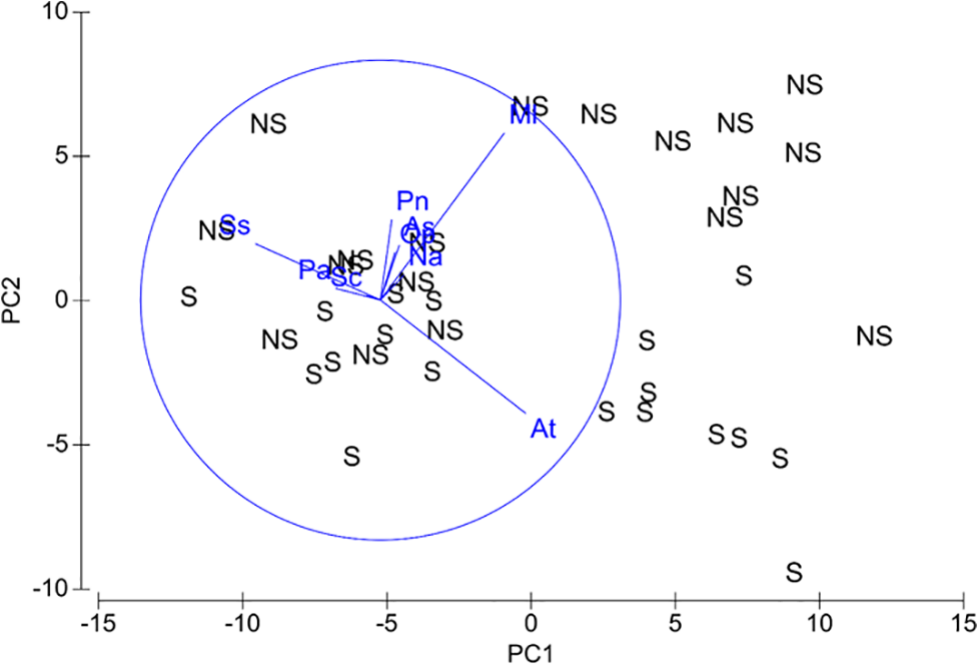
PCA ordination illustrates little effect of treatment (shaded versus non-shaded) on rates of individual ecosystems functions. Vector overlays denote different proxies of ecosystem function (all listed in Table 1 were included in the analysis, though only a selection were presented on this figure for reasons of clarity).

Ordination plots and categorical comparisons of MF in shaded and non-shaded plots did not reveal any significant effects of the shading treatment (one-way PERMANOVA, *p* = 0.76; Fig 4). None of the constituent components of the MF measure (see Table 1) differed according to the shade treatment either (one-way PERMANOVA, *p* > 0.35 in all cases). However, the analysis of the drivers of MF in shaded and non-shaded plots revealed differences in the types and numbers of significant explanatory variables and in the amount of variability explained.

The total variation in MF explained by predictor variables in the best sequential DistLM model solution (from initial ‘full’ models that included densities of several of the most common species abundances, measures of macrofaunal community richness, diversity and evenness, and sediment properties including chlorophyll-*a*, grain size and pore water nutrient concentrations) was 64% and 15% in the non-shaded and shaded treatments, respectively (Table 3). Densities of large adult *M*. *liliana* (>20 mm; measured at the plot scale) were not significantly related to MF (*p* > 0.23), but *M*. *liliana* densities derived from sediment cores dominated by individuals <5 mm shell length were the most significant predictor of MF in non-shaded sediments in both marginal and sequential tests (R^2^ = 0.24, *p* = 0.003; Table 3). Variance partitioning analysis of the non-shaded MF data (64% total explained) showed Simpson’s D’ contributing 13% and mud and pore water-NO_X_ together contributing another 24%. In contrast, in the shaded plots, *N*. *aestuariensis* was the only significant contributor to the 15% total explained variance (Table 3).

**Table 3:** Distance Based Linear Model results showing variance in MF explained by predictor variables when fitted individually (marginal tests) and in sequential models for non-shaded and shaded plots.

	Non-shaded			Shaded		
	F	*P*	R^2^	F	*p*	R^2^
**Marginal tests**						
Mud	2.06	0.111	0.11	2.18	0.070	0.12
OC	2.14	0.100	0.12	0.26	0.946	0.02
Chl-*a*	1.03	0.374	0.06	1.11	0.357	0.06
pw-NH_4_^+^	0.19	0.951	0.01	0.45	0.797	0.03
pw-NO_X_	3.67	0.036	0.19	0.22	0.925	0.01
N	4.68	0.006	0.23	1.74	0.135	0.10
Simpson’s D	1.39	0.066	0.13	0.49	0.751	0.03
*M*. *liliana* (plot)	1.46	0.225	0.08	1.11	0.349	0.06
*M*. *liliana* (cores)	5.17	0.003	0.24	1.88	0.120	0.11
*A*. *stutchburyi*	4.37	0.008	0.21	1.04	0.378	0.06
*N*. *aestuariensis*	4.44	0.008	0.22	2.92	0.023	0.15
**Sequential tests**						
*Non-shaded*						
Full model R^2^: 0.64						
Variation partitioned by *M*. *liliana* (24.4%), Simpson’s D (13.3%) and sediment properties (Mud + pw-NO_X_; 24.0%)						
*Shaded*						
Full model R^2^: 0.15						
Variation partitioned by *N*. *aestuariensis* (15.4%)						

Mud = mud content (% [>63 μm]), OC = organic content (%), Chl-*a* = chlorophyll-*a* content (μg g^−1^ sediment), pw-NO_X_ = pore-water NO_X_ concentration (μmols l^−1^), N = macrofauna community abundance (n), Simpson’s D = Simpson’s diversity index (core^−1^), all individual species abundances (n core^−1^).

## Discussion

Integrative ecological measures that capture the complexity of ecosystems are a necessity for ecosystem-based management, and those that exhibit changes in ecosystem functioning in response to subtle environmental shifts are the most urgently required [6]. Analysing changes in relationships between structural variables and processes has been suggested to provide a sensitive indicator of subtle stressor effects on ecosystem functioning [11]. More recently, Thrush et al. (2014) [34] demonstrated altered interaction strengths and losses of positive feedbacks between sediment biogeochemistry, macrofauna and microphytobenthos (MPB) in response to subtle stressor effects on sandflats. The implication of the Thrush et al. (2014) [34] study was that weakened and lost feedbacks would result in changes in ecosystem functions. This companion paper provides *in situ* empirical data on ecosystem functions from a subset of the Thrush et al (2014) [34] experimental plots.

We tested for changes in sandflat rates and processes in conjunction with a shift in light levels in a New Zealand estuary that often experiences high levels of turbidity. Importantly, there are notable differences in the manner of light attenuation by shade-cloths and that of turbidity; turbidity varies according to wind/wave action and oscillating tidal currents, and is only influential during tidal immersion (whereas shade cloths attenuate light most effectively when plots are fully exposed during low tide). Nonetheless, a change in light levels is something that would accompany further increases in estuarine turbidity levels, and was predicted to influence functioning by altering interactions involving MPB, the main primary producer in the system. Our results did not reveal any changes in the magnitudes of process variables indicative of functioning that suggested a response to the shading treatment. That is, none of the individual rates and processes (fluxes of oxygen, nitrogen and phosphorus in light and dark chambers) differed according to the shading treatment, and even the multivariate measure of functioning (MF), based on the entire suite of fluxes, did not differ by treatment. Yet, there were subtle differences in the drivers of MF between the shaded and non-shaded plots (revealed by distance-based linear models, DistLM, see below) that were consistent with the changes in system architecture demonstrated by Thrush et al. (2014) [34]. In the DistLM analysis, there were fewer significant predictors of MF in experimentally shaded plots, and less variation was explained. This suggests that, overall, interaction strengths in the system may be weakened when stressed. Moreover, the amount of variation in MF explained by total *M*. *liliana* abundance declined substantially in the shaded plots, indicating a reduced functional role for this bivalve in shade-stressed conditions. This makes sense given the presence of feedbacks connecting *M*. *liliana* and MPB [27], [29], [34], [44], [45].

The DistLM analysis also indicated that in shaded plots, where the effects of total *M*. *liliana* abundance were no longer significant, the influence of another species, *N*. *aestuariensis*, emerged (Table 3). Nereid polychaetes are highly mobile deposit feeders and predators; their gallery burrowing behaviour can play an important role in sediment mixing, irrigation and solute transport, especially when present at high densities (e.g. >300 individuals per m^2^) [46], [47]. However, in this case, the proportion of variation in MF in shaded plots explained by *N*. *aestuariensis* was small compared to the sum of biological variables explaining MF in non-shaded plots. Our results suggest that sandflat ecosystem functions are potentially susceptible to stressors affecting dominant species, though the absence of significant shifts in MF in response to the shade treatment in our study suggests that other factors and/or stressors (e.g. hydrodynamics, ambient turbidity) likely superseded the biological effects on ecosystem functioning.

In applying the shade treatment, we aimed to reduce light levels that influence key processes such as benthic primary production and nutrient efflux from sediments, to produce legacies of altered ecosystem interactions (sensu Foster et al. 2003, [48]). We found that the shading of surficial sediments had negligible effects on ecosystem properties and the magnitudes of functional response variables. For example, MPB biomass was highly variable and did not differ significantly between non-shaded and shaded treatments, potentially due to the lateral advection of surface sediments across the sandflat and under the shade cloths; this would explain the lack of shading effects on functions such as net and gross primary productivity. Nonetheless, shading appeared to modify the relationship between MPB biomass and *M*. *liliana* abundances (Fig 1), and reduced the influence of macrofauna on MF. Hydraulic pressure gradients driven by the behaviours of large *M*. *liliana* influence pore-water nutrient and oxygen dynamics [28], [29], and structural equation models suggest that shading can affect feedbacks involving *M*. *liliana*, nutrients, and MPB biomass [34]. Thus, here, the reduced significance of *M*. *liliana* densities and shifts in variables explaining variation in MF in shaded sediments (e.g. pore-water nitrate concentration no longer significant) is consistent with the loss of feedbacks described by Thrush et al. (2014) [34].

*M*. *liliana* was the numerically dominant macrofaunal species on the sandflat we studied, and *M*. *liliana* densities (assessed using sediment cores) were the strongest predictor of MF in the non-shaded treatments. Surface bioturbation and nutrient enrichment associated with small but abundant individuals (*M*. *liliana* <5 mm shell length, which dominated our samples) may have stronger effects on ecosystem functioning than previously thought [39]. However, high densities of large *M*. *liliana* have strong negative effects on the densities of juvenile conspecifics [49], [50]. Thus, the presence and therefore functional significance of juvenile bivalves may yet be very small in the presence of larger bivalves in a non-experimental context. Moreover, since disturbances may have differential effects on juveniles versus adults [51], size-class distributions and life-stages should also be accounted for when assessing stressor effects on species-function relationships [19].

To summarise, we used a multivariate metric of ecosystem function similar to that used by Villnäs et al. (2013) [35] to examine how sandflat functions responded to experimental light reduction in a physically dynamic area. We found that this stressor altered the combination of factors contributing to ecosystem function, but that functioning itself did not markedly change. This seems to suggest that functioning has a degree of resilience to changes in interaction network architecture (e.g. [13], [34]) and that higher levels of this stressor would be required to significantly alter the functioning of the system. Human activities result in multiple interacting stressors that can reinforce each other to have large ecological consequences [52]. Thus incorporating different levels of multiple stressors may be an important next step to linking changes in ecosystem functions to altered interaction networks.

## Supporting Information

S1 Table Data for sediment properties and macrofaunal abundances collected from experimental plots at the end of the experiment. Shade trt, level of shade treatment (shaded or non-shaded procedural controls); Mac trt, level of *Macomona liliana* density treatment (0, 50, 200 individuals >20 mm shell length per plot); MGS = median grain size (μm); mud, mud content (%); OC, organic content (%); Chl-a, chlorophyll-a content (μg g^−1^ sediment); Phaeo, phaeopigment content (μg g^−1^ sediment); pw-DRP, pore-water DRP concentration (μmols l^−1^); pw-NH_4_^+^, pore-water NH_4_^+^ concentration (μmols l^−1^); pw-NO_X_, pore-water NO_X_ concentration (μmols l^−1^); Ml (plot), density of large (>20 mm shell length) *M*. *liliana* per 0.25m^2^ excavated area in each plot at the end of the experiment; As (plot), density of large *Austrovenus stutchburyi* per 0.25m^2^; S, number of taxa per core; N, number of individuals per core; Shannon H, Shannon’s diversity index (core^−1^); Simpsons D, Simpson’s diversity index (core^−1^). All individual species abundances are averages from 3 cores: Ml, *Macomona liliana*; Ss, *Soletellina siliquens*; At, *Aonides trifida*; Sc, *Scolelepis* sp.; Pa, *Paphies australis*; Op, *Orbinia papilosa*; As, *Austrovenus stutchburyi*; Pn, *Paracalliope novizealandiae*; Na, *Nicon aestuariensis*; Td, *Trochodota dendyi*.
(XLSX)

S2 Table Data for process-based measures of ecosystem function derived from measures of solute fluxes across the sediment–water column interface in chamber incubations on experimental plots. NPP, net primary production; GPP, gross primary production; GPP_Chl-a_, biomass normalised gross primary production; SOC, sediment O_2_ consumption; Net NH_4_^+^, net NH_4_^+^ efflux; Net NO_X_, net NO_X_ efflux; Net DRP, net DRP efflux; NH_4_^+^ up, NH_4_^+^ uptake; NO_X_ up, NO_X_ uptake; DRP up, DRP uptake. All data given are in units of μmol solute m^−2^ h^−1^. See S1 legend for Shade Trt and Mac Trt definitions.
(XLSX)
